# Targeted versus Universal Neonatal Hearing Screening in a Single Egyptian Center

**DOI:** 10.1155/2013/574937

**Published:** 2013-09-12

**Authors:** Safaa S. Imam, Rania A. El-Farrash, Hesham M. Taha, Helbees E. Bishoy

**Affiliations:** ^1^Department of Pediatrics, Faculty of Medicine, Ain Shams University, Abbassya Square, Cairo 11381, Egypt; ^2^Audiology Unit, ENT Department, Faculty of Medicine, Ain Shams University, Abbassya Square, Cairo 11381, Egypt; ^3^Ministry of Health, Cairo, Egypt

## Abstract

*Aim*. To compare targeted neonatal hearing screening (TNHS) and universal neonatal hearing screening (UNHS) since many developing countries, including Egypt, implement selective screening for high-risk neonates. *Methods*. 150 neonates were assessed; 50 full terms consecutively admitted to the well-baby nursery and 100 neonates consecutively admitted to neonatal intensive care unit (NICU), Ain Shams University. Patients were further subdivided into high-risk group which included 50 neonates with multiple risk factors for hearing loss and low risk group which included 50 neonates with only one risk factor. Transient evoked otoacoustic emissions (TEOAEs) were used for hearing screening. Auditory brain response (ABR) was performed 3 months later for failed TEOAEs. *Results*. The most frequent risk factor was consanguinity (46%). In the well-baby population, 16% failed TEOAEs. In the NICU, 30% of the low risk and 38% of the high risk groups failed TEOAEs. Regarding ABR, failed results were 12%, 10%, and 8% in the high-risk, low-risk, and healthy groups, respectively. *Conclusion*. The use of TNHS would have missed 8% of neonates from the well-baby group who actually had PCHL (permanent congenital hearing loss). The use of UNHS would identify all cases with PCHL, allowing for early intervention and follow-up.

## 1. Introduction

Significant hearing loss is one of the most frequent congenital diseases present at birth occurring in about 1 to 3 of every 1,000 healthy neonates and 2 to 4 of every 100 neonates in the neonatal intensive care unit (NICU) population [1, 2], although the prevalence of hearing loss varies because of differences in criteria for defining hearing impairment, the age group surveyed, and the testing methods used.

Many etiological factors may result in severe hearing loss in neonates. Genetic causes account for at least 50 to 60 percent of childhood hearing loss. Environmental causes of hearing loss includes, for example, rubella embryopathy, prematurity, bacterial meningitis, and head trauma [3].

The initial signs of hearing loss are very subtle, and systematic neonatal hearing screening is the most effective means of early detection. The early identification of congenital hearing loss is necessary to minimize the consequences of hearing impairment on the future communication skills of a baby [4, 5].

The Joint Committee on Infant Hearing (JCIH) has set 3 goals: screening should be completed by 1 month of age, diagnosis should be made by 3 months, and intervention and treatment should commence by 6 months [5]. This was achieved by technologic advances in automated neonatal hearing-screening technology, resulting in the introduction of universal neonatal hearing screening (UNHS). 

Transient Evoked Otoacoustic Emissions (TEOAEs) are the most adequate hearing screening tests because they are accurate, economic, and of simple execution. In recent years, automated auditory brain stem response (AABR) instruments have been developed for screening neonates in hospital setting in order to identify patients with auditory neuropathy, and it is also important to reduce the number of false-positives [6].

 In Egypt, there is a relatively high prevalence of consanguineous marriage together with poor antenatal care and lack of public awareness about the importance of hearing screening. Therefore, despite the presence of reliable screening methods (TEOAEs, ABR) and effective methods of management, UNHS programs are still not available. On the other hand, targeted neonatal hearing screening (TNHS) or risk-based hearing screening of neonates with risk factors for hearing loss is considered a compromise between no screening at all and UNHS.

Accordingly, this study was conducted to compare the targeted, risk-based hearing screening and screening of the well-baby populations. A secondary aim was to investigate the frequency and the relative importance of each risk factor for hearing loss among the at-risk neonates.

## 2. Patients and Methods

### 2.1. Patients

This prospective cohort study was conducted at the NICU and well-baby nursery populations, Ain Shams University Hospital, between March 2009 and March 2010 on 150 neonates. One hundred consecutively admitted neonates with high-risk registry for hearing loss as listed by JCIH [5] comprised the targeted screening group or the at-risk group while 50 full-term consecutive births of non consanguineous parents, in the well-baby nursery, with no risk factors for hearing loss comprised the control group. Exclusion criteria were neonates whose admission lasted for less than 48 hours, and neonates died before the age of 3 months or did not attend an indicated ABR at 3 months. In fact, 42 out of 51 (82.3%) children with failed initial screening have attended the subsequent ABR assessment. 

All neonates were subjected to the following: detailed medical, perinatal, and family history and thorough clinical examination with an emphasis on estimation of gestational age and assessment of Apgar score at 1 and 5 minutes. Otological examination included external examination of auricles and otoscopic examination for patency and structure of external auditory canal, tympanic membrane structure, and consistency. Laboratory investigations included complete blood count (CBC) with differential leucocytic count using Coulter Counter GEN-S (Beckman Coulter, USA). Diagnosis of hyperbilirubinemia was done by measuring total serum bilirubin level using Hitachi 911 Analyzer (Boehringer, Germany) and interpretation of the results according to the age-specific nomogram [7] which recommends responses ranging from careful monitoring to phototherapy or, ultimately, exchange transfusion. 

#### 2.1.1. Group Classification

 Neonates were classified into 3 groups; group A (high-risk group) included 50 neonates with multiple (3-4) risk factors for hearing loss; group B (low-risk group) included 50 neonates with only one risk factor, and group C (control group) included 50 healthy neonates. 

#### 2.1.2. Neonatal Hearing Screening

 It was done by the same observer (H. M. Taha, MD) using a two-staged TEOAEs screening tests, where initial TEOAEs were done immediately after birth followed by a retest prior to hospital discharge, and then failures of this second stage were referred for the diagnostic ABR assessment after 3 months. Referral was done through a follow-up card given to the parents. The study protocol was approved by the local Ethical Committee of Ain Shams University.

### 2.2. Methods

#### 2.2.1. Transient Evoked Otoacoustic Emissions (TEOAEs)

 The TEOAEs were elicited using click stimuli with intensity ranging between 79 and 90 dB SPL with a mean of 83 ± 3 dB SLP using a portable TEOAEs screener, vivosonic integrity 500. Better stimulus levels were associated with better probe fit and quiet state of the infant. The software determined the amplitude of the TEOAEs in five frequency bands: 1, 1.5, 3, 4, and 5 KHz.

 Testing was done while infants were naturally sleeping or were alert and calm (no sedatives were used). If during the test infants were active or crying, the test could not be completed, and retesting was performed on another day. The results of TEOAEs were interpreted according to the Rhode Island criteria [8]; pass: response is 3 dB or more in at least 4 frequency bands, partial pass: positive response in at least 2 frequency bands, and fail: no response is present in any frequency band. Retesting was done for infants before hospital discharge, and those with failed or partial pass response in one or both ears for the second time were considered as failed screening and referred for diagnostic ABR. 

#### 2.2.2. Auditory Brain Stem Response (ABR)

 Recordings were done in the Audiology Unit, Ain Shams University Hospitals, using the evoked response audiometry Ampliad, model MK 12. Infants were naturally sleeping. Three surface electrodes were placed so that the active electrode was applied to the forehead and the reference and ground electrodes to the ipsilateral and contralateral mastoids, respectively. Acoustic rarefaction unfiltered clicks of 0.1 msec. duration were presented at a rate of 21 p/sec. at 90 dB nHL and down to threshold. At least two recordings were obtained for each intensity. A total of 1024 sweeps were differentially amplified through a band pass filter of 300–3000 Hz with 10 msec. analysis time. 

 Assessment of an ABR pass was based on the detection of “wave V” at its expected latency (5.4 milliseconds) according to our norms and down to threshold (30 dB nHL). Patients with elevated thresholds underwent low probe tone (256 Hz) tympanometry. Neonates with type (B) tympanometry received medical management until the resolution of middle ear effusion and ABR was retested when immittancemetry revealed type (A) or (C) tympanogram. Abnormal results of ABR were interpreted as Permanent congenital hearing loss (PCHL) with failed test of a ≥40 dB nHL in the presence of normal middle ear function.

 A recall of the diagnosed neonates for reevaluation was done using air and bone conduction ABR. For bone conduction, ABR bone vibrator was placed on the mastoid with effective masking applied to the other ear while testing.

### 2.3. Statistical Analysis

The data were recorded, coded, entered, and processed on computer using SPSS computer software (version 15). Comparison between categorical variables was performed using chi-square (*χ* ^2^) test or Fischer's exact test when frequencies were below five. Significant risk factors of hearing loss were entered into a multivariate analysis to determine the magnitude of the association between these factors and hearing loss and identify its odds ratio. A *P* value < 0.05 was considered significant in all analyses.

## 3. Results

Table 1 represents the descriptive statistics of the study groups. Most of the studied neonates were delivered by caesarian section (CS); 35/50 (70%) in low-risk and 34/50 (68%) in high-risk groups. Moreover, 31/50 (62%) of neonates in control group were delivered by CS and stayed for longer duration in the well-baby nursery than those who were delivered vaginally which gives us time to perform two-staged hearing screening tests. 

Table 2 shows the distribution of risk factors among the targeted-screening groups. The most frequent risk factor was consanguinity (46%) followed by mechanical ventilation (42%), very low birth weight (40%), ototoxic drugs (25%), sepsis (23%), low Apgar score (16%), and hyperbilirubinemia (12%). Stigmata of syndromes that are known to be associated with deafness accounted for 8% of cases in the targeted-screening groups; 3 neonates with intestinal atresia, 2 with trachea esophageal fistula, and 3 had Down's syndrome. Other less frequent factors include positive family history of hearing loss (2%) and craniofacial abnormalities (1%). The most frequent risk factor for hearing loss among high-risk neonates (Group A) was mechanical ventilation for more than 5 days (41/50) followed by birth weight less than 1500 gm (27/50), whereas consanguinity was the most frequent risk factor for hearing loss (25/50) among neonates of the low-risk group (Group B) followed by birth weight less than 1500 gm (13/50).

Table 3 demonstrates that 19 in the high-risk group, 15 in the low-risk group, and 8 in the control group failed the TEOAEs bilaterally, and these 42 neonates were referred for diagnostic ABR. Regarding ABR results, 12% of the high-risk group and 10% of the low-risk group had PCHL. Interestingly, 8% of neonates of the well-baby population suffered from PCHL with the number needed to screen (NNS) of 33. In other words, to detect those 4 neonates, we have to screen approximately 132 healthy neonates.

Table 4 examined the association between different risk factors and hearing tests' outcomes. None of the risk factors were significantly related to PCHL (*P* > 0.05). Multivariate analysis of the most important risk factors for hearing loss revealed that all factors were non significantly associated with impaired ABR results (*P* > 0.05).

Table 5 revealed that 11 out of the 15 children diagnosed with PCHL were able to come to the follow-up visit; 45.4% suffered from severe degree of sensorineural hearing loss (SNHL), 27.3% suffered from moderate degree of SNHL, and 27.3% suffered from mild degree of SNHL.

## 4. Discussion

Hearing loss is twenty times more prevalent in neonates than other disorders that are routinely screened for, including hypothyroidism, phenylketonuria, and sickle cell anemia. Until just a decade ago, late diagnosis was the norm, with the average age at identification of congenital hearing loss reported as 2.5 to 3 years or even later [6]. A valuable window of opportunity for early intervention was being lost. Intervention in the first 6 months of life is particularly important for speech and language development; accordingly parents and professionals overwhelmingly prefer early diagnosis [2, 5]. 

 In the current analysis, TEOAEs test revealed failed response in 42/150 (28%) infants bilaterally. Failed results were 38%, 30%, and 16% in the high-risk, low-risk, and healthy groups, respectively. The results of TEOAEs screening varied widely in the literature according to the characteristics of the screened population; In Egypt, El-Gamal et al. [9] examined 90 NICU cases and reported a failure rate of 54% in the multiple risk factors group and 20% in the single risk factor group, while Abdullah et al. [10] found that 11.8% of the screened high-risk neonates in Malaysia failed the TEOAEs test. This prevalence of failure among the healthy neonates was far higher than that cited by Korres et al. [6] who examined hearing in well-nursery babies with TEOAEs in Greece and found a failure rate of 2.3%. 

It was assumed that failure in the first hearing screening using TEOAEs in this study might be secondary to external ear obstruction and/or middle ear effusion. That is why our hearing screening protocol associated TEOAEs with otoscopic examination and external ear cleaning or suction when necessary. Additionally, two-staged TEOAEs test was performed in order to reduce the false positives. This protocol was in agreement with other studies [6, 11]. On the other hand, Clarke et al. [12] suggested that screening for hearing loss in neonates should be performed 4 days after birth. Therefore, failure in the neonatal hearing screening test must be understood as a possible hearing loss, and the diagnosis should be confirmed with the conventional ABR three months later after complete myelination of the auditory pathway [12].

It is very important to know the exact numbers of neonates who failed screening and actually had hearing loss confirmed in subsequent assessments. This is a weakness of most similar studies in the literature. However, in the current study, diagnostic ABR confirmed that bilateral PCHL in 15 neonates out of the 42 (36%) neonates failed TEOAEs screening. 

The reason for such high PCHL rate in our study could be attributed to the characteristics of the studied group of neonates admitted to the NICU at a University Hospital that is a regional excellence center and therefore provides care to highly complex cases with an expected higher prevalence of hearing loss in the population treated at this facility. Attempts to screen school children for hearing loss in Egypt, mostly secondary to middle ear conditions, documented a higher prevalence of hearing loss than that in the developed countries [13–15].

Consanguineous marriage is common in some developing communities including Egypt. It could be considered as a risk factor for hearing loss because of unrecognized higher prevalence of genes responsible for heridofamilial hearing loss [16]. In this field, Mohamed et al. [17] disclosed a novel, functionally relevant GJB2, a gene encoding the gap junction protein connexin 26 (Cx26), mutation and defined the contribution of Cx26 alterations to the hearing loss in the Southern Egyptian population. This finding suggests that among the SNHL of unknown etiology, those of a hereditary origin should be considered. With the advances in the field of molecular genetics, new genes responsible for SNHL have been identified, and the mechanisms involving nonsyndromic SNHL have been explained.

Nevertheless, there is a consensus that the levels of PCHL are greater in underdeveloped countries, with Davidson et al. [18] estimating that SNHL is twice as common. The World Health Organization [19] in its report on chronic diseases views the process from poverty to chronic diseases as “interconnected in a vicious cycle,” as poor people have greater exposure to risks and decreased access to health services. There is some impetus for an increased effort of identification of PCHL in developing countries; trials of UNHS at immunization clinics have been undertaken in Nigeria and South Africa and were successful in terms of coverage, but the attendance at follow-up was poor [20].

Another finding worth discussing in the current analysis is that if hearing screening was limited to the at-risk neonates, 4/50 (8%) neonates from the well-baby group who had actually failed the ABR test would have been missed. This finding suggests that UNHS is far preferable because many hearing impaired infants do not have any risk factors. Thus, UNHS is strongly needed putting in mind not the cost of the screening but the cost it would be to rehabilitate a deaf-mute child.

Traditionally, hearing screening has been targeted to high-risk populations which represent less than 10% of births and can potentially identify between 50% and 59% of those with congenital hearing loss. In the literature, Wroblewska-Seniuk et al. [21] reported the presence of hearing impairment in 24.9% of the high-risk registers. Moreover, de Capua et al. [22] reported 11.65% failure rate of TEOAEs among 532 examined infants of whom only 84 were high-risk registry.

Although risk factors for hearing loss have been used for screening neonates in many countries worldwide, there is no consensus with regard to the importance of each of these factors and the level of relative risk. In the present study, we tried to define prospectively the risk demarcators that could predict hearing impairment in at-risk neonates. However, no significant interaction was found between any of the studied risk factors and the impaired ABR results. 

Risk factors for hearing loss are constantly refined by the JCIH [5]; however, these factors should not be considered as the “gold standard” with the same relative importance because the situation in different countries or time periods may vary considerably [6]. For example, a developing country like ours may use more ototoxic agents without assessing drug blood levels because of the lack of resources, whereas another developed country may not use such agents. In contrast, neonates who could have died as a result of severe prematurity and birth complications in a remote center with limited funding and equipment may very well have survived in a large NICU. Therefore, continuous investigation of the relative importance of high-risk factors of hearing loss is essential to assess and refine these factors and modify accordingly current clinical practice. Moreover, such studies are very useful when funding is limited and screening is inevitably targeted to the most vulnerable neonates.

Finally, the potential limitations inherent in this study include lack of a standard method to track children who failed screening tests and missed diagnostic ABR. However, we believe that our findings provide new insights into neonatal hearing screening in a developing country like Egypt.

In conclusion, universal neonatal screening for congenital hearing loss would detect more cases than targeted, risk-based screening, allowing for early intervention. Designing hearing screening policy, refining risk factors, and implementing follow-up and treatment protocols are of paramount importance in Egypt. 

## Figures and Tables

**Table 1:** Demographic characteristics of the examined neonates.

	Group A (high-risk group) *n* = 50	Group B (low-risk group) *n* = 50	Group C (control group) *n* = 50
Sex			
Male	23	26	26
Female	27	24	24
Maturity			
Preterm	32	13	0
Full term	18	37	50
Mode of delivery			
VD	19	15	16
CS	31	35	34

VD: vaginal delivery; CS: cesarean section.

**Table 2:** Distribution of risk factors for hearing impairment among at-risk neonates.

Variable	Targeted screening *n* = 100	Group A (high-risk group) *n* = 50	Group B (low-risk group) *n* = 50
	*n* (%)	*n* (%)	*n* (%)
Consanguinity	46 (46)	21 (42)	25 (50)
Mechanical ventilation ≥ 5 days	42 (42)	41 (82)	1 (2)
Birth weight < 1500 gm.	40 (40)	27 (54)	13 (26)
Ototoxic drug	25 (25)	25 (50)	0 (0)
Sepsis	23 (23)	23 (46)	0 (0)
Low Apgar score	16 (16)	14 (28)	2 (4)
Hyperbilirubinemia	12 (12)	9 (18)	3 (6)
Stigmata with risk of deafness	8 (8)	4 (8)	4 (8)
Family history of hearing loss	2 (2)	0 (0)	2 (4)
Craniofacial abnormality	1 (1)	1 (2)	0 (0)
In utero infection	0 (0)	0 (0)	0 (0)

**Table 3:** Comparison between the results of TEOAEs and ABR of each of the risk groups with the control group.

	Group C (control)	Group A (high-risk group)		Group B (low-risk group)	
	*n* (%)	*n* (%)	*P*_1_	*n* (%)	*P*_2_
TEOAEs					
Pass	42/50 (84)	31/50 (62)	**0.013**	35/50 (70)	0.096
Fail	8/50 (16)	19/50 (38)		15/50 (30)	
ABR					
Pass	4/8 (50)	13/19 (68)	0.365	10/15 (67)	0.435
Fail	4/8 (50)	6/19 (32)		5/15 (33)	

TEOAEs: transient evoked otoacoustic emissions; ABR: automated brain stem response; *P*_1_: probability value upon comparison between group A and control; *P*_2_: probability value upon comparison between group B and control.

**Table 4:** Incidence of hearing loss among neonates with different risk factors.

Variable (*n*)	Normal	PCHL	*P*
	*n* (%)	*n* (%)	
Consanguinity (46)	43 (93)	3 (7)	0.56
Mechanical ventilation ≥ 5 days (42)	36 (86)	6 (14)	0.27
Birth weight < 1500 gm (40)	37 (92)	3 (8)	0.76
Ototoxic drug (25)	21 (84)	4 (16)	0.28
Sepsis (23)	19 (83)	4 (17)	0.25
Low Apgar score (16)	14 (87)	2 (13)	0.66
Hyperbilirubinemia (12)	10 (83)	2 (17)	0.34
Stigmata with risk of deafness (8)	6 (75)	2 (25)	0.18
Family history of hearing loss (2)	2 (100)	0 (0)	1.00
Craniofacial abnormality (1)	1 (100)	0 (0)	1.00
In utero infection (0)	0 (0)	0 (0)	—

PCHL: permanent congenital hearing loss.

**Table 5:** The degree of hearing loss among patients who came at the follow-up visit.

	PCHL cases who came at the follow-up visit (*n* = 11)		
	Mild hearing loss (*n* = 3)	Moderate hearing loss (*n* = 3)	Severe hearing loss (*n* = 5)
Group A (high-risk group)	1	1	2
Group B (low-risk group)	1	1	2
Group C (control group)	1	1	1

PCHL: permanent congenital hearing loss.
